# Validation of Various Pan‐ApoE and Isoform‐Specific ApoE Antibodies

**DOI:** 10.1002/alz.091634

**Published:** 2025-01-03

**Authors:** Dahlia Siano, Lesley R Golden, Steven M MacLean, Gabriela Hernandez, Lance A Johnson

**Affiliations:** ^1^ University of Kentucky, Lexington, KY USA; ^2^ Sanders‐Brown Center on Aging, Lexington, KY USA

## Abstract

**Background:**

Apolipoprotein E (ApoE) exists in three protein isoforms: E2, E3, and E4, which differ by only one or two amino acids. These slight differences profoundly effect protein structure and function, allowing each isoform to differentially impact Alzheimer’s Disease (AD) risk. Relative to the most common E3 isoform, E4 dramatically increases risk, while E2 confers a substantial decrease in risk. The close similarity between protein isoforms makes it difficult to develop isoform‐specific antibodies that are reliable and selective. Here, we aim to validate and optimize a number of common, commercially available ApoE antibodies to determine isoform specificity.

**Method:**

Control samples included plasma and brain collected from APOE knockout (KO), homozygous E2, E3 and E4 humanized APOE (hAPOE) mice, and human plasma from all 6 possible *APOE* genotypes. Western blotting was used on plasma and brain homogenates to determine isoform specificity of E2, E3, or E4 antibodies. Commercial antibodies tested included pan‐ApoE antibodies from Cell Signaling Technologies (CST) and Abcam, ApoE4 antibodies from Novus and CST, ApoE2 antibodies from CST, and ApoE3 antibodies from Abcam and Novus. Additionally, pan‐ApoE antibodies were kindly gifted to us by collaborators (Drs. Lammich and Haass, LMU) and tested. Antibodies were also tested for use in immunohistochemistry (IHC) using hAPOE and APOE KO mouse brain sections (30 uM).

**Result:**

Despite the ApoE3 antibodies being marketed as isoform‐specific, they did not show efficacy in either application. Commercial pan‐ApoE antibodies were outperformed by the LMU antibodies, but still showed efficacy. When used for western blotting, the CST ApoE4 and ApoE2 antibodies showed clear isoform specificity in hAPOE mouse and human samples. While none of the isoform‐specific antibodies passed controls for IHC purposes, multiple pan‐ApoE antibodies were variably effective for staining of brain tissue.

**Conclusion:**

These data identify several isoform‐specific ApoE antibodies that are reliable and selective across multiple sample types and applications; albeit some display higher affinity for one application over another. Overall, we hope these results will provide AD researchers with important resources that are crucial for visualizing and quantifying ApoE isoform distribution in heterozygote individuals and across a number of AD models, systems, and studies.

